# Barriers and enablers to exclusive breastfeeding by mothers in Polokwane, South Africa

**DOI:** 10.3389/fgwh.2024.1209784

**Published:** 2024-02-13

**Authors:** Maishataba Solomon Makwela, Reneilwe Given Mashaba, Cairo Bruce Ntimana, Kagiso Peace Seakamela, Eric Maimela

**Affiliations:** ^1^ Department of Public Health, Faculty of Health Sciences, University of Limpopo, Polokwane, South Africa; ^2^ Department of Human Nutrition and Dietetics, Faculty of Health Sciences, University of Limpopo, Polokwane, South Africa; ^3^ DIMAMO Population Health Research Centre, University of Limpopo, Polokwane, South Africa

**Keywords:** breastfeeding, feeding practices, factors, barriers, enablers

## Abstract

**Background:**

Exclusive breastfeeding (EBF) for six months, with the introduction of appropriate complementary feeding thereafter, and breastfeeding continuing for up to 2 years and beyond, is highly recommended. This could save the lives of up to 1.4 million children each year worldwide. Despite this, breastfeeding rates in South Africa remain sub-optimal, with the recommended target of 50% by the World Health Assembly (WHA) not being achieved. The study aimed to investigate the reasons influencing mothers' practice of exclusive breastfeeding in the Polokwane municipality of Limpopo province in South Africa.

**Methodology:**

A cross-sectional health facility-based quantitative and descriptive survey was conducted using a validated-structured questionnaire administered to 146 mothers. The data was analyzed using STATA. Chi-square tests were used to determine the relationship between selected demographic variables and their reasons not to breastfeed exclusively.

**Results:**

Although 94% of the mothers had initiated breastfeeding, at the time of data collection 8% had stopped. Of those who had stopped breastfeeding, 5% did so within one month of starting. Thirty- nine percent of mothers' breastfed exclusively, while 61% practiced mixed feeding. A positive association between exclusive breastfeeding practices and the age of the mother were observed, with older mothers more likely to breastfeed. The reasons mothers stopped breastfeeding were: the mother was ill (45%) or they returned to school or work (27%). Reasons for not breastfeeding were cited as: medical conditions, not enough milk, and infant refusal to breastfeed (33%). Mothers believe that HIV-positive women should breastfeed their infants (57%), and health workers were found to be the main source of HIV information to mothers (77%).

**Discussion:**

Exclusive breastfeeding during the first six months was less practiced. Infant formula and solid foods were introduced at an early age, usually within the first month of breastfeeding. This study sheds light on factors influencing the early initiation of breastfeeding and the practice of EBF as practiced in Polokwane.

## 1 Introduction

Breastfeeding is the process of feeding the infant with the mother's milk (1). It is an integral part of the reproductive process with important implications for the health of the mother and baby (2). The feeding of an infant with only breast milk and no additional food, water, or other liquids (except for medicines and vitamins, if needed) during the first six months of life is termed exclusive breastfeeding (EBF)) (3–5). This is the feeding practice for newborns and infants recommended by the World Health Organization (WHO) to reduce infant morbidity and mortality (5–7). EBF is considered superior to the mixed infant feeding method which includes a combination of breastmilk and infant formula and/or other food types (8). The importance of counselling as a strategy to improve breastfeeding practices has been highlighted in the United Nations Children's Fund (UNICEF) and WHO guidelines to improve breastfeeding. The United Nations (UN) also established targets to eliminate malnutrition and increase exclusive breastfeeding (EBF) rates to at least 50% (9, 10). It is recommended that breastfeeding should start within one hour of birth, this helps the infant with all nutrients necessary for growth from colostrum. Colostrum reduces child morbidity and mortality (1, 11).

EBF should be practiced for up to six months and breastfeeding should continue until the child is two years of age (4, 11). EBF is recommended because breast milk contains all the nutrients necessary in the first few months of life (11). Breast milk is the best nutrition for preterm infants in optimizing the growth and development of babies and young children and is an effective intervention for preventing early childhood deaths (2, 5). It is posited that EBF could prevent 1.4 million deaths worldwide among children under the age of five every year if implemented (5).

Even though EBF is the most efficacious type of infant feeding, women around the world still fail to reach the recommended target of 6 months postpartum (1, 7). Furthermore, sub optimally practiced breastfeeding, negatively affects child survival, growth, and development (1, 7). Infants less than two months old who are not breastfed are six times more likely to die from diarrhea and acute respiratory infections than their counterparts (11, 12). Those who are not exclusively breastfed have a high chance of acquiring pneumonia, meningitis, ear infections and dying from childhood-related diseases due to inadequate Timely Initiation of Breastfeeding (TIBF) (11, 12).

Mothers have numerous challenges which may influence their choice to breastfeed. These may include; a perceived lack of breastmilk, cracked or sore nipples, breast engorgement, disapproval and discomfort of breastfeeding in public, and insufficient breastfeeding support from society and healthcare providers (13). In addition, short maternity leave periods for working mothers, difficulties associated with combining breastfeeding and other maternal responsibilities, and emotional stress are also part of the challenges faced by mothers which may negatively affect their choice to breastfeed (12, 13). The South African government has committed itself to improving EBF, but there is a lack of information on reasons why mothers are not exclusively breastfeeding in rural areas of Limpopo (14). This study aimed to investigate the prevalence of EBF and barriers and enablers of EBF among mothers attending primary health care clinics around Polokwane municipality, Limpopo province.

## 2 Materials and methods

### 2.1 Study design, population, and sampling

The study was cross-sectional in design and conducted in the Capricorn District of the Limpopo Province in northern South Africa. The municipality consists of townships, suburbs, and villages within an area of 3,766 km^2^ and an estimated population size of 797,127, of which 2% are white, 97% African, and 1% are colored [“(15): District Municipality: Capricorn”]. The majority of residents of this district are Sepedi speaking (84.9%) followed by 2.97% Afrikaans, 2.60% Xitsonga, 2.03% English, 1.99% isiNdebele, 1.60% other, 1.41 Tshivenda, 1.05% isiZulu, 0.77 Sesotho, 0.67 Setswana [“(15): District Municipality: Capricorn”]. Polokwane Municipality has an estimated sex ratio of 92.8 males per 100 females.

In this study, a total of five primary health care (PHC) facilities were randomly selected, while the community health Centre (CHC) was automatically included as it was the only referral site within its cluster. The participants were selected using convenient sampling. The study consisted of 146 mothers, paired with their infants from a group of approximately 491 infants who attended six primary health care (PHC) facilities every month in Polokwane, South Africa.

The study participants were informed about the possibility of publishing the findings of the study. The study was approved by the Turfloop Research Ethics Committee (TREC) with reference TREC/115/2017: PG under the University of Limpopo. The Department of Health (DoH) in Limpopo Province granted permission for the study to be conducted in the PHC and CHC facilities. All mothers who participated in the study did so after signing a written full informed consent. In cases where the mother was under age (<18 years) the research team sought consent from them and their legal guardian or parents.

#### 2.1.1 Inclusion criteria

The inclusion criteria for the study were as follows:

• Mothers of infants aged 0–6 months who visited the facility for immunization on the days of data collection
• Mothers who gave written consent to take part in the study.

#### 2.1.2 Exclusion criteria

The exclusion criteria for the study were as follows:

• Infants brought in by caregivers who are neither biological mothers nor primary caregivers
• First-time mothers with infants zero to two weeks – because they may have limited infant feeding experience
• Mothers and caregivers of children over six months of age
• Mothers with an infant with any kind of metabolic disease or genetic disease.

### 2.2 Data collection

A structured questionnaire was used to collect data adapted from Goosen et al. (16). This was to answer a set of closed-ended questions that sought information about the mothers and infants. The questionnaire included questions on socio-demographic information, infant feeding practices, knowledge of caregiving, and medical information. To test for the face validity of the tool, a pilot study was conducted. The questionnaire was piloted at one of the selected primary health care facilities and the results from the pilot study were used to adjust the questionnaire accordingly.

### 2.3 Statistical analysis

Data were analyzed using STATA statistical software (STATA Corporation, College Station, Texas) version 12. Categorical variables are presented as percentages and continuous variables are expressed as mean ± SD. *χ*^2^ tests were used to evaluate relationships between different selected variables. The critical value for significance was set at *p* < 0.05 for all analyses. Descriptive statistics were used to summarize the socio-demographic characteristics of the study population, the prevalence of infant feeding practices, and factors influencing these practices. Binary logistic regression was used to determine the association between exclusive breastfeeding and associated factors.

### 2.4 Results

Table 1 describes the demographic characteristics of the mothers who participated in the study. Out of the 146 mothers who participated in the current study, the majority of participants (54.1%) were in the age category 26–35 years. All participants aged ≤18 years were still in high school. Overall, 65.8% of the participants were unemployed with the age group of 26–35 years having the highest percentage of both employment status categories; employed (72%) and unemployed (48.9%). About 33.2% of the participants were first-time mothers. Approximately 53% were receiving child support grants and older mothers were more likely to be receiving child support grants.

**Table 1:** Demographic characteristics of the mothers.

	Overall	Age categories of mothers				*p*-value
		<18 years	18–25 years	26–35 years	>35 years	
Total *n* (%)	146 (100.0)	8 (5.5)	40 (27.4)	79 (54.1)	19 (13.01)	
Education						
Primary *n* (%)	2 (1.4)	–	–	2 (100)	–	0.164
High *n* (%)	58 (39.7)	7 (12)	19 (32.8)	27 (46.6)	5 (8.6)	
Matric *n* (%)	40 (27.4)	1 (2.5)	9 (22.5)	23 (57.5)	7 (17.5)	
Tertiary *n* (%)	46 (31.5)	–	12 (26.1)	27 (58.7)	7 (15.2)	
Employment status						
Employed *n* (%)	50 (34.2)	–	8 (16)	36 (72)	6 (12)	0.006
Unemployed *n* (%)	96 (65.8)	–	32 (36.4)	43 (48.9)	13 (14.8)	
Birth order						
Firstborn *n* (%)	53 (36.3)	8 (15.1)	29 (54.7)	16 (30.2)	–	<0.001
Second born *n* (%)	44 (30.1)	-	10 (22.7)	32 (72.7)	2 (4.5)	
Third born *n* (%)	26 (17.8)	-	1 (3.8)	20 (76.9)	5 (19.2)	
Fourth born *n* (%)	16 (11.0)	–	–	10 (62.5)	6 (37.5)	
Fifth born *n* (%)	7 (4.8)	–	–	1 (14.3)	6 (85.7)	
Support grant						
Yes *n* (%)	78 (53.4)	5 (6.4)	21 (26.9)	43 (55.1)	9 (11.5)	<0.001
No *n* (%)	68 (46.6)	3 (4.4)	19 (27.9)	36 (52.9)	10 (14.7)	

Table 2 shows the association of demographics with infant feeding practices. There was no association between the sex of the child and exclusive breastfeeding and, as expected exclusive breastfeeding decreased with the increased age of the infant (*p* = 0.065; insignificant) and was less significantly practiced by mothers of the age category of >35 years (*p* = 0.036). Most infants were delivered at public health facilities (84.22%), followed by those delivered in private health facilities (14.4%) and those delivered at home (1.4%) (*p* = 0.254). The present study found a significant association between an increase in the age of the mother with exclusive breastfeeding (*p* = 0.036), where older mothers were found to exclusively breastfeed when compared to younger mothers. In the age group <18 years, those who practiced mixed feeding were 87% as compared to 13% of EBF. In the age group 18–25 years, those who practiced mixed feeding were 70% as compared to 30% of EBF. In the age group 26–35 years, those who practiced mixed feeding were 58% as compared to 42% of EBF. On the contrary, in the age group >35 years, those who practiced mixed feeding were 37% as compared to 63% of EBF. EBF was practiced less by mothers who delivered at private health facilities (28.6%) when compared to those who delivered at public health facilities (42.3%). Although not significant, 32% of employed mothers practiced EBF and the results of the current study indicate that mothers who were full or part-time employed practiced less EBF. Even though it was not statistically significant, mothers with high school as their highest level of education practiced EBF less.

**Table 2:** Association of mother-infant paired demographics with infant feeding practice.

	Total *n* (%)	Exclusive breastfeeding practices		*p*-value
		Exclusive breastfeeding *n* (%)	Not exclusively breastfeeding n (%)	
Breastfeeding practices		53 (39)	84 (61)	
Infants gender				
Boy *n* (%)	75 (51.4)	30 (40)	45 (60)	0.945
Girl *n* (%)	71 (48.6)	28 (39.4)	43 (60.6)	
Age of infant				
0–1 month *n* (%)	7 (4.8)	5 (71.4)	2 (28.6)	0.065
1–3 months *n* (%)	67 (45.9)	30 (44.8)	37 (55.2)	
4–6 months *n* (%)	72 (49.3)	23 (31.9)	49 (68.1)	
Place of infant birth				
Public clinic/hospital *n* (%)	123 (84.2)	52 (42.3)	71 (57.7)	0.254
Private clinic/hospital *n* (%)	21 (14.4)	6 (28.6)	15 (71.4)	
Home	2 (1.4)	0 (0)	2 (100)	
Mothers age				
<18 years *n* (%)	8 (5.5)	1 (13.0)	7 (87.0)	0.036
18–25 years *n* (%)	40 (27.4)	12 (30.0)	28 (70.0)	
26–35 years *n* (%)	79 (54.1)	33 (42.0)	46 (58.0)	
>35 years *n* (%)	19 (13.0)	12 (63.0)	7 (37.0)	
Mothers employment status				
Employed *n* (%)	50 (34.2)	16 (32.0)	34 (68.0)	0.169
Unemployed *n* (%)	96 (65.8)	42 (43.8)	54 (56.2)	
Level of education				
Primary school *n* (%)	2 (1.4)	1 (50.0)	1 (50.0)	0.835
High school *n* (%)	58 (39.7)	21 (36.2)	37 (63.8)	
Matric *n* (%)	40 (27.4)	18 (45.0)	22 (55.0)	
Tertiary education *n* (%)	46 (31.5)	18 (39.1)	28 (60.9)	
Support grant				
Yes *n* (%)	78 (53.4)	29 (37.2)	49 (62.8)	0.501
No *n* (%)	68 (46.6)	29 (42.6)	39 (57.4)	

Table 3: Table 3 shows that approximately 94.5% of mothers had received advice on how to feed their infants, of which 69% had received this advice during pregnancy. Significantly more mothers (58%) who received breastfeeding advice were not exclusively breastfeeding (*p* = 0.018). Approximately 5% of mothers had not received advice on infant feeding and all these mothers did not exclusively breastfeed their infants. The majority (65.6%) of the mothers reported that they had received infant feeding information during pregnancy compared to 30.4% who received it after delivery. About 92.7% of mothers started breastfeeding their babies immediately after birth. Those who delayed breastfeeding stated reasons such as the mother's illness (57.1%), and infant illness (38.8%) with the last reason being lack a lack of knowledge on how to start breastfeeding (4.1%). Mothers who gave their infants non-prescribed medication were significantly more likely to practice EBF (69.4%) (*p < *0.001). Reasons for giving non-prescribed medicines included perceived attributes of the medication to make the infant grow well (10.1%), prevent diseases (81.7%), treat umbilical cord/fontanelle (5.5%), and prevent crying/colic (2.8%) with no significant difference between those who practiced exclusive breastfeeding and those who did not.

**Table 3:** Barriers and enablers to the practice of exclusive breastfeeding.

	Total	Infant feeding practices		*p*-value
		Exclusive breastfeeding	Not exclusive breastfeeding	
Received advice on infant feeding				
Yes *n* (%)	138 (94.5)	58 (42.0)	80 (58.0)	0.018
No *n* (%)	8 (5.5)	0 (0.0)	8 (100)	
Timing of feeding advice				
During pregnancy *n* (%)	96 (69.6)	43 (44.8)	53 (55.2)	0.320
After delivery *n* (%)	42 (30.4)	15 (35.7)	27 (64.3)	
Infant ever breastfed				
Yes *n* (%)	137 (93.8)	79 (57.7)	58 (42.3)	0.012
No *n* (%)	9 (6.2)	0 (0.0)	9 (100)	
Received help to start breastfeeding				
Yes *n* (%)	80 (58.4)	48 (60.8)	32 (55.2)	0.512
No *n* (%)	57 (41.6)	31 (39.2)	26 (44.8)	
Start of infant breastfeeding				
Immediately *n* (%)	127 (92.7)	71 (89.9)	56 (96.6)	0.267
2 h after birth *n* (%)	8 (5.8)	6 (7.6)	2 (3.5)	
3–6 h after birth *n* (%)	2 (1.5)	2 (2.5)	0 (0.0)	
Reasons for not breastfeeding after 1 h				
Mother ill *n* (%)	28 (57.1)	15 (48.4)	13 (72.2)	0.193
Infant was ill *n* (%)	19 (38.8)	15 (48.4)	4 (22.2)	
Didn't know how to start breastfeeding *n* (%)	2 (4.1)	1 (3.2)	1 (5.6)	
Should HIV + mothers breastfeed				
Yes *n* (%)	83 (56.9)	36 (62.1)	47 (53.4)	0.579
No *n* (%)	55 (37.7)	19 (32.8)	36 (40.9)	
Not sure *n* (%)	8 (5.4)	5 (5.1)	5 (5.7)	
Give infants non-prescribed medicines				
Yes *n* (%)	110 (75.3)	25 (69.4)	11 (30.6)	<0.001
No *n* (%)	36 (24.7)	33 (30.0)	77 (70.0)	
Reasons to give non-prescribed medicines				
Infant grow well *n* (%)	11 (10.1)	8 (10.5)	3 (9.1)	0.552
Prevent diseases *n* (%)	89 (81.7)	60 (78.9)	29 (87.9)	
Treat umbilical cord/fontanelle *n* (%)	6 (5.5)	5 (6.6)	1 (3.0)	
Prevent crying/colic *n* (%)	3 (2.8)	3 (3.9)	0 (0.0)	

Table 4: At the time of the interview, eleven mothers, which is 8% of the participants who had initiated breastfeeding at one time, had stopped breastfeeding. Mothers who stopped breastfeeding indicated the following reasons; illness (45.5%), going back to school (27.3%), and not producing enough milk (18.2%). Mothers who did not breastfeed (never initiated breastfeeding) indicated the following reasons, illness/medical condition (33.3%), and not enough milk (33.3%), and infant refused to be breastfed 220 (33.3%). About 85.7% of mothers gave infants solids food and liquids mainly because they felt they could not produce enough milk and the infants were crying. Most mothers received their infant feeding information from health workers (33.3%), and media (25.4%), and 5.7% of the mothers received the feeding information from their families. Approximately 0.7% of the participants reported that breastfeeding promotes bonding between mother and infant. The most frequently reported reason for breastfeeding was the perception that breast milk was the perfect nutrition/food for infants, followed by that breast milk is free at 33.6% and 30.0% respectively.

**Table 4:** Reasons behind feeding choices.

Reasons for stopping breastfeeding	Frequency *N* (%)
I was ill/with an infectious disease	5 (45.4)
Going back to work/school	3 (27.3)
Not enough milk	2 (18.2)
Breastfeeding conditions	1 (9.1)
Reasons for not breastfeeding (Never initiated breastfeeding)	
Mother was ill/had a medical condition	3 (33.3)
Not enough breast milk	3 (33.3)
The infant refused to be breastfed	3 (33.3)
Reasons for giving solids and liquids	
Not enough milk/infant crying/Breast milk not enough	60 (85.7)
Advice from a family member	4 (5.7)
Advice from a healthcare worker	3 (4.3)
Infant hungry/infant crying/Infant crying/breast milk not enough/advice from a family member	2 (2.9)
Not enough milk	1 (1.4)
Source of infant feeding information	
Media	35 (25.4)
Health worker/media	23 (16.7)
Family/health worker	16 (11.6)
Family	8 (5.7)
Family/media	6 (4.3)
Family/health worker/media	4 (2.9)
Reasons For Breastfeeding	
Breastfeeding is perfect for babies	46 (33.6)
Breastfeeding is the perfect nutrition for babies/OTHER	41 (30.0)
Breastfeeding is perfect for babies/free of charge	11 (8.0)
Other	9 (6.6)
Breastfeeding is perfect for babies/infants who are ill	5 (3.6)
Free of charge/no need to prepare/OTHER	5 (3.6)
Breastfeeding is perfect for babies/free of charge/free of charge/infant was ill/no need to prepare	4 (3)
No need to prepare/OTHER	1 (0.7)
Bonding with the infant	1 (0.7)
Breastfeeding is perfect for babies/free of charge/no need to prepare	3 (2.2)
Breastfeeding is perfect for babies/Free of charge/OTHER	3 (2.2)
Free of charge	3 (2.2)
Breastfeeding is perfect for babies/infants who are ill/OTHER	3 (2.2)
Breastfeeding is perfect for babies/no need to prepare	2 (1.4)
Sources of advice for giving men prescribed medication	
Family member	95 (65.1)
Health worker	10 (6.8)
Family/health worker	5 (3.4)
Did not give un-prescribed medication	36 (24.7)

Table 5: Binary logistic regression showed that mothers aged >35 years were 12.0 times more likely to practice EBF (*p* = 0.034)*.* Infants aged 4–6 months were 0.2 times less likely to be exclusively breastfed (*p* = 0.026). Women who were not first-time mothers (*p* = 0.029), unemployed (*p* = 0.011)*,* and mothers who received help (*p* = 0.639) to start breastfeeding were more likely to practice EBF. Mothers who did not receive advice on infant feeding (*p* = 0.018), those who received advice on breastfeeding after delivery (*p* = 0.033), and those who gave their infant non-prescribed medications (*p* < 0.001) were less likely to practice exclusive breastfeeding.

**Table 5:** Binary logistic regression for determinants of exclusive breastfeeding.

Variables	Exclusive breastfeeding		
	OR	95% CI	*p*-value
Age of mother			
<18 years	Reference		
18–25 years	3.0	0.33–27.12	0,328
26–35 years	5.0	0.58–42.78	0.140
>35 years	12.0	1.21–118.88	0.034
Age of infant			
0–1 month	Reference		
1–3 months	0.3	0.06–1.79	0.197
4–6 months	0.2	0.03–1.04	0.026
First-time mother			
Yes	Reference		
No	1.7	0.82–3.39	0.029
Employment			
Employed	Reference		
Unemployed	1.3	0.98–1.75	0.011
Level of education			
Tertiary education	Reference		
Primary school	1.6	0.09–26.47	0.760
High school	0.9	0.39–1.96	0.760
Matric	1.3	0.53–3.00	0.582
Received advice on infant feeding			
Yes	Reference		
No	0.4	0.07–0.70	0.018
Received help to start breastfeeding			
Yes	Reference		
No	1.2	0.60–2.29	0.639
Timing of getting advice on breastfeeding			
During pregnancy	Reference		
After delivery	0.7	0.32–1.44	0.033
Should HIV + mothers breastfeed			
Yes	Reference		
No	0.7	0.34–1.39	0.301
Not sure	0.8	0.17–3.49	0.749
Give infants non-prescribed medicines			
No	Reference		
Yes	0.2	0.08– 0.43	<0.001

## 3 Discussion

The prevalence of EBF in the present study was 39%, which is higher than the 32% national prevalence (17). However, the findings of the present study are similar to those reported in a study by du Plessis et al (18),, which reported a prevalence of 38.5% (18). On the other hand, a study conducted in the North West province, South Africa, reported a low prevalence of EBF at 9.7% in the infant age group of 20–24 weeks (19). The difference may be due to different study settings, sample sizes, EBF policies and practices, and cultural norms. Given the WHO and UNICEF targets of reaching 50% of EBF at a national level by 2025, the strong points of policies, practices, and cultural norms of countries with a high prevalence of EBF could be used to strengthen our policies and practices to meet the WHO and UNICEF targets (19). Whilst the recorded increase in exclusive breastfeeding rates for children under 6 months is positive, the continuing disparities across different reports create uncertainty about the actual progress made (20).

The major reason women gave for not practicing EBF (mixed feeding prevalence 61%) in the current study was that they perceived that they did not have enough milk. Similar findings were reported in a survey conducted in the Eastern Cape, South Africa (21). The survey found that early introduction of food, water, and infant formula milk was common despite a high breastfeeding initiation rate (21). Similarly, Goosen et al. (16), attributed the early introduction of food and water to the mother's perception that the infants needed water or other supplements (16). On the other hand, the reasons for discontinuation of breastfeeding in the current study were that the mothers were ill, returning to either school or the workplace, and breast conditions such as which concurs with other studies (22, 23). In addition, women who are affected by post-natal depression and related mental health challenges stemming from socioeconomic challenges and lack of support were reported to have a higher likelihood to stop breastfeeding in less than three months postpartum (19). On the other hand, the main reasons given by mothers in the current study for breastfeeding their infants were that breastmilk provides adequate nutrition for the infant and that breastmilk is free. Similarly, a study by Modjadji et al. (17), reported breastfeeding as economical and convenient, promoting child growth and development, protecting the infant against illness, and promoting mother-baby bonding (17). Breast milk contains all the nutrients an infant needs, and promotes child survival, health, brain, and motor development (24, 25).

Evidence shows that the prevalence of breastfeeding, including EBF declines as infants get older (6). An increase in the age of the mother was found to be an indicator of EBF in the present study, where older mothers exclusively breastfed their infants when compared to younger mothers. The Binary logistic regression showed that mothers aged >35 years were 12.0 times more likely to practice EBF. In accordance with the present study, a study conducted in the USA by Jones et al. also reported that the mother's age was strongly associated with the likelihood of breastfeeding exclusively for 6 months (26). Young mothers do not exclusively breastfeed their infants due to their busy lifestyles, being at school, and lack of confidence in breastfeeding (27). In the current study, the prevalence of EBF was high in younger infants (71.4% below 1 month) and it was much lower (31.9%) in infants aged between 3 and 6 months. These rates are still far below the targeted EBF rates of 50% (28, 29). Similarly, the low prevalence at 3–6 months was reported in a study conducted by Joshi et al. (4).

The majority of employed mothers did not practice EBF and the results of the current study indicate that mothers who were full or part-time employed practiced less EBF. In binary logistic regression, unemployed mothers were more likely to practice EBF. This finding is in accordance with the findings of a study conducted by Mandal et al., in the United States of America, which found that full-time employment status was negatively correlated with breastfeeding initiation and duration, suggesting that full-time employment remains a significant barrier to breastfeeding (30). Mothers in a study in Cape Town, South Africa, cited the lack of breastfeeding facilities in public spaces and at work as reasons for failure to exclusively breastfeed their infants (31). In addition, employed mothers face challenges such as the need to return to work early for economic reasons and an unfavorable work environment for expressing and storing milk which makes EBF a challenge for them (32).

In the current study, 64.4% of mothers initiated breastfeeding within one hour after delivery. The initiation rate in the current study is lower compared to the global target of 80% but similar to the national prevalence of 67% in 2016 (33). Initiation of breastfeeding in the first hour post-delivery is recommended as one of the interventions that have the potential to improve infants' nutritional outcomes and reduce infant mortality (34, 35). Early initiation of breastfeeding is associated with sustainable breastfeeding for up to 2 years and beyond (36–38). In the present study, failure to initiate breastfeeding within the first hour was mostly attributed to the mother's illness. Other mothers reported separation from their infants as a cause of delayed initiation as supported by Mukunya et al. (39), who also found a significant association between separation of the mother-baby pair and delayed breastfeeding initiation (39).

Another reason for the delayed initiation of breastfeeding in the current study was a lack of knowledge of how to initiate breastfeeding. Lack of breastfeeding counseling and midwife support has been previously identified as a cause for increased delay in the initiation of breastfeeding (39, 40). These authors emphasize the importance of breastfeeding education during antenatal visits and support by midwives and birth attendants post-delivery to ensure early breastfeeding initiation. Khanlari et al. (41), reported early breastfeeding initiation among mothers who received support from birth attendance immediately after delivery (41). Other challenges contributing to the lack of breastfeeding knowledge include the discrepancies in the knowledge of breastfeeding by health professionals in relation to WHO recommendations (42). A study by Zweigenthal et al. (42), highlighted the need for ongoing training in breastfeeding and infant feeding counseling, as well as community-based postnatal support initiatives addressing cultural beliefs as a way to promote knowledge of breastfeeding amongst mothers (42).

Even though only about two-thirds of mothers initiated breastfeeding within an hour post-delivery, about 94% of the mothers did initiate breastfeeding at some point post-delivery. Studies indicate that it is a common practice for most mothers to initiate breastfeeding (43, 44). Even though the rate of breastfeeding declines as the infants get older (45). These findings are consistent with the results of a study conducted by Siziba et al. (46) in four provinces of South Africa, which reported breastfeeding initiation at 90% (46). Mothers who were not first-time mothers were more likely to exclusively breastfeed. In agreement with the present study, a study by Balogun et al. (47), reported that first-time mothers with no prior experience of breastfeeding were less likely to practice exclusive breastfeeding, while women with at least one child had a higher likelihood of practicing EBF (47). First-time mothers are likely to lack experience in breastfeeding and knowledge of how to initiate it which could be the reason why in the present study mothers who are not first-time mothers were more likely to practice EBF. In addition, a study by Lutaaya et al. (48), reported the following factors as barriers to EBF in first-time mothers; mothers' perception of babies needing more than milk had the highest percentage, followed by fear of breasts losing shape, having difficulties in EBF, breastfeeding being old fashion and maternal understanding of EBF and its recommended period (48). Mothers who did not receive advice on how to initiate breastfeeding were less likely to practice EBF. Similar findings were reported by Massare et al. (49).

## 4 Conclusion

Exclusive breastfeeding during the first six months was less practiced. Infant formula and solid foods were introduced at an early age. This study sheds light on factors influencing the early initiation of breastfeeding following delivery and the practice of EBF as practiced in Polokwane.

## 5 Recommendations in light of key study findings

Given that the present study reported a low prevalence of EBF in comparison to the WHO and UNICEF goals the present study recommends that mothers be educated on the importance of EBF, particularly young ones. The study shows that there is a high use of traditional medicine among mothers; therefore, there is a need to properly implement the South African Traditional Health Act (No. 22 of 2007) with the aim of regulating the prescription of traditional medicine. It would also be beneficial to the country to speed up a review of labor regulations to increase maternity leave to six months to afford mothers more time for breastfeeding. Workplace support for breastfeeding will be more effective if it includes lactation breaks and breastfeeding rooms. Given that older mothers practice exclusive breastfeeding for a longer duration than younger mothers, there is a need to raise awareness of the significance of exclusive breastfeeding among young mothers.

## Data Availability

The raw data supporting the conclusions of this article will be made available by the authors, without undue reservation.
